# A cross‐sectional analysis of Alzheimer’s disease plasma biomarkers and gait variables in cognitively normal older adults

**DOI:** 10.1002/alz.091941

**Published:** 2025-01-09

**Authors:** Wajiha Ahmed, Allal Boutajangout, Yuekun (Hillary) Shao, Sophia Madjarova, Ziyan Ma, Ludovic Debure, Jon Links, Sohail Karimi, Alok Vedvyas, Karyn Marsh, Rebecca A Betensky, Thomas Wisniewski, Arjun V. Masurkar

**Affiliations:** ^1^ NYU Alzheimer's Disease Research Center, New York, NY USA; ^2^ Center for Cognitive Neurology, New York University Langone Health, New York, NY USA; ^3^ NYU Grossman School of Medicine, New York, NY USA; ^4^ Center for Cognitive Neurology, New York, NY USA; ^5^ New York University, New York, NY USA; ^6^ Alzheimer's Disease Research Center, New York University Langone Health, New York, NY USA

## Abstract

**Background:**

A decline in gait has been associated with an escalated risk of cognitive decline and changes in Alzheimer’s disease (AD) biomarkers, thus offering prognostic insight. However, the utility of gait analysis in preclinical stages of AD is unclear, and prior studies have primarily used qualitative or gross measures of gait. Furthermore, gait analysis has predominantly been performed in cohorts of non‐Hispanic Whites. We analyzed the correlations between plasma biomarkers and quantitative gait variables in a diverse cohort of cognitively normal older adults.

**Method:**

A cohort of 129 consecutive participants (age 56‐93, 30% Black/ African American, 10% Hispanic/Latino) from the NYU Alzheimer’s Disease Research Center were confirmed cognitively normal via the Uniform Data Set Version 3 psychometric battery. Participants underwent quantitative gait analysis via the GAITRite walkway. Means and intra‐individual variability (expressed as standard deviation across the laps) of gait variables were assessed over the eight passes of the walk. Plasma levels of Aβ42, Aβ40, NfL, pTau181, and total tau were determined using Quanterix SIMOA. The associations between the gait measures and plasma biomarkers were analyzed using multivariable linear regression models, adjusting for age, sex, and ApoE status.

**Result:**

The mean velocity showed a statistically significant negative association with NfL and total tau, after adjusting for age, sex, and ApoE genotype. A 1pg/ml increase in NfL was associated with a 0.33 unit decrease in mean velocity (p=0.028). A 1pg/ml increase in total tau was associated with a 3.53 decrease in mean velocity (p=0.013). Intra‐individual variability in gait velocity also showed a statistically significant positive association with pTau181. A 1pg/ml increase in ptau181 level showed a 0.385 unit increase in velocity variability (p=0.018).

**Conclusion:**

AD plasma biomarkers show correlations to quantitative gait measures even in a cognitively normal population, apart from age and ApoE genotype. Our findings also promote the utility of both means and variability of gait measures in stratifying preclinical risk of AD.

